# A value framework for lymphoma therapies based on MACBETH method

**DOI:** 10.1017/S0266462325000169

**Published:** 2025-05-19

**Authors:** Yumei He, Wei Li, Xiaochen Zhu, Zhifeng Nie, He Zhu, Yingyao Chen, Sheng Han

**Affiliations:** 1 International Research Center for Medicinal Administration, Peking University, Beijing, China; 2 Department of Pharmacy Administration and Clinical Pharmacy, School of Pharmaceutical Science, Peking University, Beijing, China; 3 School of Public Health, Fudan University, Shanghai, PR China; 4 National Health Commission Key Laboratory of Health Technology Assessment, Fudan University, Shanghai, PR China

**Keywords:** MCDA, MACBETH method, Lymphoma, Healthcare decision-making, value assessment framework

## Abstract

**Objectives:**

The rising cost of oncology care has motivated efforts to quantify the overall value of cancer innovation. This study aimed to apply the MACBETH approach to the development of a value assessment framework (VAF) for lymphoma therapies.

**Methods:**

A multi-attribute value theory methodological process was adopted. Analogous MCDA steps developed by the International Society for Health Economics and Outcomes Research (ISPOR) were carried out and a diverse multi-stakeholder group was recruited to construct the framework. The criteria were identified through a systematic literature review and selected according to the importance score of each criterion given by stakeholders, related research and expert opinions. The MACBETH method was used to score the performance of alternatives by establishing value functions for each criterion and to assign weight to criteria.

**Results:**

Nine criteria were included in the final framework and a reusable model was built: quality adjusted life years (QALYs), median progression-free survival, objective response rate, the incidence of serious adverse events (grade 3–4), rates of treatment discontinuation due to adverse events, annual direct medical costs, dosage and administration, the number of alternative medicines with the same indication and mechanism, mortality of the disease. The weights of each criterion in the order presented above are 17.43 percent, 16.11 percent, 14.39 percent,13.54 percent,11.83 percent,11.30 percent,7.08 percent,4.59 percent, and 3.73 percent.

**Conclusions:**

A criterion-based valuation framework was constructed using multiple perspectives to provide a quantitative assessment tool in facilitating the delivery of affordable and valuable lymphoma treatment. Further research is needed to optimize its use as part of policy-making.

## Introduction

In recent years, the cost of cancer therapy has been rising as new therapies are being presented in the clinic. However, the additional clinical benefits of these expensive new cancer drugs are probably limited. One study showed that the available evidence for 125 drugs (58 percent) out of the 216 new drugs approved for the market in Germany between 2011 and 2017 did not prove an added benefit over standard care for mortality, morbidity, or health-related quality of life in the approved patient population (1). The increasing spending on healthcare technologies and limited clinical benefits of new drugs prompted growing efforts in exploring value-based assessment models.

To support decision-making, several healthcare-related and scientific societies, including the American Society of Clinical Oncology (ASCO), the National Comprehensive Cancer Network (NCCN), the European Society for Medical Oncology (ESMO), Memorial Sloan Kettering Cancer Center (MSKCC) have launched frameworks designed to assess the value of oncology therapies. Multi-criteria Decision Analysis(MCDA) has been widely applied in health care and oncology decision-making. Drug value assessment frameworks based on MCDA have been established across different disease areas, including colorectal cancer, rare diseases, diabetic macular edema, and other disease areas according to published studies outside China (2–4).

MACBETH (Measuring Attractiveness by a Categorical Based Evaluation Technique) is an MCDA approach, based on pairwise qualitative comparisons, using qualitative judgments about the difference of attractiveness between different pairs of attribute levels (5;6). Semantic judgments made either by individuals or groups are converted into a cardinal scale, providing a simple, constructive, and interactive approach with good prospects for facilitating the preference elicitation process of groups (7). MACBETH(measuring attractiveness by a categorical-based evaluation technique)method has strong theoretical foundations (8), numerous applications for real-world problems (7;9), and is expected usefulness in HTA settings.

In China, the theories and methodologies of MCDA have been applied to various practices including the drug bidding and procurement process, the drug selection for the Essential Medicine List, and the evaluation of clinical therapies. However, in general, the application of MCDA in China’s health care system as a policy tool is still in the initial and exploratory stage.

Lymphoma (including Hodgkin’s lymphoma and non-Hodgkin’s lymphoma) is one of the most common diseases that threaten public health. China has approximately one-fifth of the world’s population and faces a dramatic disease burden of lymphoid neoplasms (10). To our knowledge, no drug value evaluation tool in Lymphoma has been constructed in China. Therefore, we used the MACBETH method to construct a value assessment framework for lymphoma drugs, providing a model for value assessment in this field.

## Methods

We constructed the value framework with the following steps, which were adjusted according to MCDA steps developed by ISPOR (11;12): (1) defining the decision problem, (2) selecting criteria, (3) constructing value functions, (4) weighting criteria, (5) testing consistency. The detailed methods by step are as follows:

### Defining the decision problem

To establish a reusable value framework for lymphoma therapeutics from a medical insurance payer perspective in China.

### Selecting criteria

Criteria were established through a literature review and stakeholder interviews. First, we summarized the current value framework criteria for oncology drugs. A systematic review of value frameworks for oncology drugs in PubMed, EMbase, Web of Science, VIP database(China), Wanfang database(China), and China National Knowledge Infrastructure (CNKI) was undertaken. An example of the search strategy used in PubMed is shown in Supplementary Appendix 1. Additionally, value frameworks published on the official web sites of ASCO, ESMO, NCCN, MSKCC, ICER, CADTH, and PPVF were reviewed.

Subsequently, we surveyed 15 stakeholders (3 physicians, 7 pharmacists, 3 health economists, and 2 medical insurance experts) from Beijing, Shanghai, Ningxia, Shandong, and Fujian provinces to determine the importance of the criteria from the literature review. The background of the stakeholders selected was referred to the panel of review experts of The National Healthcare Security Administration, who were responsible for the adjustments of the National Reimbursement Drug List. The stakeholders were asked to give a score between 0 and 5 for each criterion, with 0 being the least important and 5 being the most important. Based on the survey responses, criteria with an average score of ≤3.5 were excluded. The questionnaire is shown in Supplementary Appendix 2.

Since the criteria finally would be used in an additive model, the remaining criteria need to meet the following five requirements: completeness, non-redundancy, nonoverlap, preference independence, and operability (11).

### Constructing value functions

MACBETH approach was used to construct value function with M-MACBETH software.

We designed a questionnaire (shown in Supplementary Appendix 3) according to the attractiveness difference judgment matrix in the M- MACBETH software. In this step, how to set the Performance Reference Levels for each criterion is a key issue. The more the number of performance reference levels is, the more accurate the function will be. However, too many reference levels could increase the difficulty of understanding and affect the reliability and validity of the questionnaire. Therefore, we set five reference levels for each criterion. The setting of performance reference levels was based on drug information collected from a systematic literature review of relevant real-world studies, key clinical trials supporting the drug launch, drug specifications, burden of disease studies, and pharmacoeconomic studies related to lymphoma therapeutics launched in China from 2017 to 2021. For the incidence of serious adverse events (grade3–4) and the treatment discontinuation rate due to adverse events (AE-TDR) indicators, the lowest value of collected drug performance data is regarded as “Level 2,” the highest value is regarded as “Level 4,” and the median value is regarded as “Level 3.” The lower 20 percent of the lowest value is regarded as “Level 1,” and the higher 20 percent of the highest value is regarded as “Level 5.” For other indicators, the highest value of collected drug performance data is regarded as “Level 2,” the lowest value is regarded as “Level 4,” and the median value is regarded as “Level 3.” The upper 20 percent of the highest value is regarded as “Level 1,” and the Lower 20 percent of the lowest value is regarded as “Level 5.” The performance levels are shown in Table 1.

**Table 1.** Five performance reference levels for each criterion

Performance reference level		mPFS (month)	QALYs	ORR	≥3 AE	AE-TDR	ADMC (RMB)	Dosage and administration	Mortality (per 100,000)	The number of alternative medicines
Level 1		49	11.5	100%	17%	2%	128000	Oral, once a day	2.94	0
Level 2		41	9.6	93%	21%	3%	160000	Oral, twice a day	2.45	1
Level 3		17	5.2	79%	58%	10%	200000	Intravenous injection, every three weeks	1.32	2
Level 4		5	1.8	35%	91%	25%	390000	Intravenous injection, every two weeks	0.19	3
Level 5		4	1.4	28%	100%	30%	468000	Intravenous injection, once a week	0.15	4

ADMC, annual direct medical costs.

We selected 28 stakeholders (7 phyicians, 7 pharmacists, 7 health economists, 7 medical insurance experts) from Beijing, Shanghai, Tianjin, Sichuan, Fujian, Henan, Shandong, Guangdong, and Liaoning provinces and asked them to pairwise compare the attractiveness difference between each performance reference level above. The background of the stakeholders selected was referred to the panel of review experts of The National Healthcare Security Administration, who were responsible for the adjustments of the list of medicines covered by the medical insurance system.

### Weighting criteria

The weight of each criterion was obtained by a MACBETH procedure through a qualitative swing weighting approach. It qualitatively judged differences in the attractiveness of a set of referential, hypothetical alternatives. The hypothetical alternatives consist of “lower” and “upper” performance reference levels preset for each criterion. Hypothetical alternatives are shown in Supplementary Appendix 4.

Stakeholders were asked to compare the overall attractiveness differences of the hypothetical schemes in Supplementary Appendix 4 pairwise. After the consistency test, weights are generated for each criterion.

### Testing consistency

A consistency check between the qualitative judgments expressed was automatically provided by M-MACBETH software, and a second consistency check was performed manually by the facilitator to ensure that an interval scale is obtained, i.e., validate the cardinality of the scale (7).

Intraclass correlation coefficient (ICC) was used to evaluate the inter-rater reliability of questionnaires which informed the consistency check results. ICC value is between 0 and 1, where 0 indicates untrusted and 1 indicates fully trusted. It is widely believed that a reliability coefficient lower than 0.4 indicates poor reliability, whereas a reliability coefficient greater than 0.75 indicates good reliability.

## Results

### Criteria

Twenty five criteria through literature review were ranked according to experts’ scoring results as follows: median overall survival, annual direct medical costs, health-related quality of life, improvement in tumor-related symptoms, clinical irreplaceability, median progression-free survival, the treatment discontinuation rate due to adverse events, objective response rate, complete response, cost-utility, duration of response, the incidence of Serious Adverse Event (grade 3–4), unmet clinical needs, the severity of disease, treatment-free interval, Tail of the Curve, innovation in therapeutic mechanisms, changes in drug delivery modalities, sequence of clinical treatments, budget impact, prevalence, burden on caregivers, equity, increase in social productivity, and incidence of adverse events(grade 1–2).

Firstly, the last seven criteria were excluded based on the principle that the mean expert score is larger than 3.5 points. Secondly, based on the principle of data availability and non-redundancy, we excluded the following criteria: (1) median overall survival, improvement in tumor-related symptoms, duration of response, and treatment-free interval are rarely reported in clinical trials, which makes data difficult to obtain; (2) CR was excluded because it was almost never used as a primary efficacy endpoint in clinical trials and had similar meaning with the higher-ranked criteria ORR; (3) cost-utility, which includes concepts of total cost and quality of life; (4) unmet clinical needs, for no official definition and quantitative evaluation method; (5) tail of the curve, because it is influenced not only by the efficacy of the drug but also by other reasons such as the length of follow-up, sample size, and different treatments after progression; (6) innovations in therapeutic mechanisms, which is difficult to quantify. Thirdly, we made the following adjustments according to data availability: (1) we used QALYs as a measurement for health-related quality of life. QALY is a comprehensive index that combines the quality of life and length of life; (2) we used “dosage and administration” represents “Changes in drug delivery modalities.” (3) “severity of disease” was represented by “mortality of disease.” (4) clinical irreplaceability was measured by the number of alternative medicines with the same indication and mechanism. The selection process can be found detailed in Supplementary Appendix 5.

**Figure 1.**
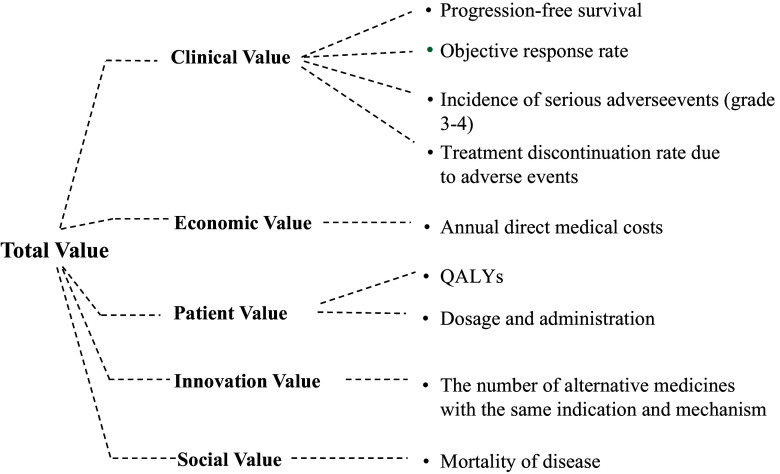
Value assessment framework (VAF) for lymphoma therapies.

Finally, nine criteria were included: progression-free survival (PFS), objective response rate (ORR), incidence of serious adverse events (grade3–4), treatment discontinuation rate due to adverse events (AE-TDR), quality-adjusted life years (QALYs), annual direct medical costs (ADMC), dosage and administration, mortality of disease, the number of alternative medicines with the same indication and mechanism. The nine criteria were presented in the form of value tree (see Figure 1).

### Value function

**Figure 2.**
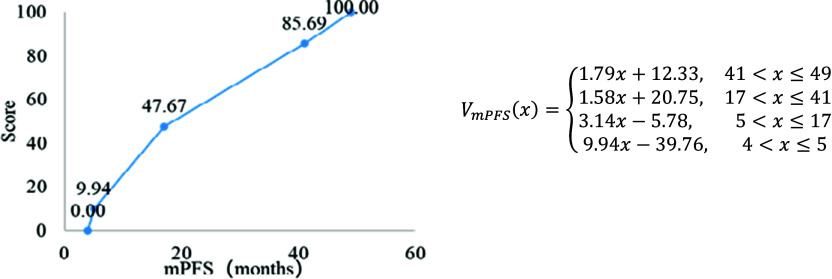
Piecewise linear value function of mPFS.

The piecewise linear value functions of mPFS are shown in Figure 2, and the corresponding formula are detailed below the figure. Due to space limitations, the piecewise linear value functions of other criteria are shown in Supplementary Appendix 6. Among them, mPFS, QALYs, ORR, serious adverse events (grade 3–4), AE-TDR, ADMC, and Severity of disease are numerical variables, represented in curve form. Although feasibility and innovation are categorical variables, represented in scatter plot form.

With these value functions, if the performance of lymphoma therapies on each criterion can be found, the score for each criterion can be calculated.

For example, if mPFS of drug A is 25.7 months, based on Figure 2 the value score of drug A in the mPFS criterion is calculated as 61.356, with the formula as follows:

VmPFS (25.7) = 1.58 × 25.70 + 20.75 = 61.356.

### Weight

**Figure 3.**
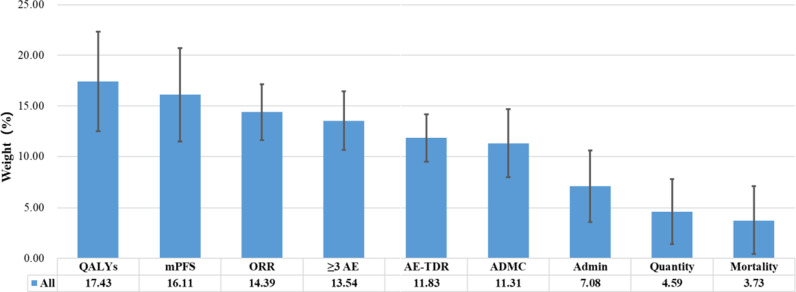
Mean weight results of all stakeholder evaluations.

The mean weight results of all stakeholder evaluations are shown in Figure 3. From Figure 3, we can see that QALYs take the largest weight of 17.43 percent, followed by mPFS and ORR which takes 16.11 percent and 14.39 percent respectively. Results from all stakeholders show that criteria related to quality of life, effectiveness, and safety take larger weight. Mortality of disease takes the smallest weight, which is 3.73 percent.

**Figure 4.**
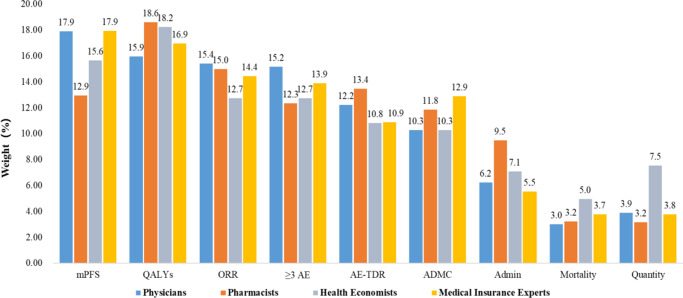
The criterion weight ranking results from different types of stakeholders.

Different stakeholders may rank the importance of criteria differently, so we present the criterion weight ranking results from different types of stakeholders (Figure 4). Physicians, Pharmacists, Health economists, and Medical insurance experts all believed that the weight ratio of mPFS, QALYs, and ORR ranked in the top three, whereas the order was different. In addition, health economists believed that serious adverse events (Grade3–4) took the same weight as ORR, ranking third. To our surprise, the largest weight from medical insurance experts was mPFS rather than QALYs. To the best of our knowledge, medical insurance pays for QALYs according to current Chinese policy. Adverse events did not rank among the top three importance in Pharmacists’ opinions.

### Inter-rater reliability

A total of 28 questionnaires were sent out with a response rate of 100 percent, and the inter-rater reliability was found to be good (ICC, 0.944; 95 percent CI, 0.916–0.966).

## Discussion

Rapidly growing cancer drug prices give rise to resource allocation issues calling for consideration of value for money. Drug value evaluations have become increasingly important when new cancer treatments are launched to the market. Drug value evaluations should consider multiple dimensions and criteria. Therefore, we adopted a MACBETH approach, which has been used in published research (3) to provide a comprehensive assessment of the value of Lymphoma therapeutics.

The value assessment framework is a promising tool for measuring the value of health technologies and informing the policy-making of drug coverage. It is important to identify high-value drugs for the medical insurance list considering the budget constraint. Value framework could be applied to evaluate the value of drugs both inside and outside the medical insurance, which is conducive to the dynamic adjustment of the National Reimbursement Drug List. Besides, decision-makers in hospitals with a limited procurement budget would also find drug value assessments useful with clearly defined criteria, scientific methods, and transparent procurement processes.

MACBETH method is able to illustrate the association between the performance on a given criterion and the preference for that performance in a much transparent manner by constructing value functions for each criterion. Through MACBETH procedure, we were also able to develop a reusable model to assess new alternatives with more evidence available. Our study provides a hands-on quantitative assessment tool for the value evaluation of lymphoma therapeutics and further enriches health technology assessment studies using MCDA method in China.

Finally, we constructed a value framework consisting of nine criteria, involving the preferences of key stakeholders from four fields including clinical, medical insurance, pharmacy, and health economics. The essence of our study is to construct a multi-criteria decision analysis model. There is currently no rule as to how many criteria should be included in an MCDA analysis (11). A recent review of MCDAs in health care found that an average of 8.2 criteria were used to assess interventions, with the number of criteria ranging from 3 to 19 (11). ISPOR MCDA Good Practice Guidelines suggested that it is good practice to have as few criteria as is consistent with making a well-founded decision, though the analyst should consider the trade-off between an increase in validity from a more complete set of criteria and the potential for reducing the validity of scores or weights as a result of the time and cognitive effort associated with more criteria (11).

Currently, there is no specific value assessment framework for lymphoma treatments both domestically and internationally. However, there are value assessment frameworks for the whole oncology treatments, including those from ASCO, NCCN, ESMO, MSKCC, and the oncology value assessment procedure developed by CADTH.

The ASCO Value Assessment Framework was established in 2015 and updated in 2016. The scoring system primarily includes three aspects: clinical benefits, toxicity, and bonus points. Two versions of the framework have been developed: one for advanced cancer and another for potentially curative treatment. The sub-criteria of clinical benefit is ranked as mOS, mPFS, and RR. If data on median OS are not available, median PFS data are to be used instead. Using advanced disease framework provides an opportunity to receive bonus points in cancer-related symptom (or palliation bonus), treatment-free interval, improvement in QoL, and tail of the curve (13).

The NCCN Value Framework was established in 2015, focusing on five value dimensions: efficacy, safety, quality of evidence, consistency of evidence, and affordability (14).

The ESMO Value Assessment Framework was released in 2015 and updated in 2017 (15). ESMO-MCBS considers clinical benefit (PFS and OS, both absolute gain and hazard ratio (HR)), toxicity (Grade 3–4 toxicities assessment), and QoL (disease-free interval, event-free survival, time to recurrence, PFS, and time to progression), etc. (15;16).

MSKCC developed the MSKCC-DrugAbacus/Drug Pricing Lab, an interactive computational program that can be used online to evaluate the value of anti-cancer drugs. The value assessment tool includes eight criteria: survival impact, toxicity, scientific novelty, cost of development, rarity, population burden, need unmet, and prognosis (17).

The Canadian Agency for Drugs and Technologies in Health (CADTH) developed the pan-Canadian Oncology Drug Review (pCODR), released in 2011, which aims to assess new anti-cancer drugs and/or new clinical indications. The pCODR Expert Review Committee has established an evaluation framework (pERC deliberative framework), including overall clinical benefit (effectiveness, safety, burden of illness and need), alignment with patient values, cost-effectiveness (economic evaluation, costs, cost per QALY, cost per life year gained, cost per clinical event avoided, uncertainty of net economic benefits) and feasibility of adoption into the health system (economic feasibility-budget impact assessment, organizational feasibility) (18).

The value frameworks of ASCO, NCCN, ESMO, MSKCC, and CADTH both prioritize clinical efficacy and safety. Similarly, in the framework developed in this study, efficacy and safety indicators also carry significant weight, aligning with existing value frameworks. Additionally, the value framework constructed in this article is relatively comprehensive and representative, with value dimensions not considered in current frameworks, such as innovation and severity of disease.

## Limitations

This study entails several limitations as well. First, in the selection process of criteria, we excluded criteria such as unmet clinical needs, treatment-free interval, and improvement in tumor-related symptoms from the initial list. These criteria may be important but either cannot be quantified, lack clarity in definition, or are difficult to obtain data. Cost-utility was also excluded to avoid double counting, considering this measurement could capture values of multiple aspects, including cost, efficacy, safety, quality of life, etc. However, the final criteria list of our value framework is comprehensive enough, for it reflects the most important values considered in China’s major medical decision-making. Currently, in China, the inclusion of new drugs into the national reimbursement drug list is mainly decided by evidence on safety, efficacy, economy, innovation, and equity. These factors are all covered by the criteria listed in our study. Second, the value framework developed in this study is from a medical insurance payer perspective, which is applicable to the adjustment of the National Reimbursement Drug List in China. Stakeholders included in this study are experts involved in the adjustment of the National Reimbursement Drug List in China, representing a comprehensive set of recommendations from physicians, pharmacists, health economists, and medical insurance experts. Last, inherent to all MCDA, the limited number of stakeholders may not represent the opinion of all the actors involved. Moreover, the weights and scores assigned in this MCDA reflect the perception of the stakeholders on the current exercise, for this reason, the external validity of the results will not be evident. In the same way, it must be considered that the criteria of the MCDA are based on the experience, knowledge and value judgments of the stakeholders. Hence, the analysis contains certain subjectivity.

## Conclusion

In this study, a criterion-based valuation framework for lymphoma therapies was designed using multiple perspectives. It’s an important step toward the improvement of drug affordability and the delivery of high-value lymphoma care in China. Further research is needed to optimize its use as part of policy-making.

## Supporting information

He et al. supplementary material He et al. supplementary material

## Supplementary material

The supplementary material for this article can be found at http://doi.org/10.1017/S0266462325000169.

## References

[1] Wieseler B, McGauran N, Kaiser T. New drugs: Where did we go wrong and what can we do better? BMJ. 2019;366:l4340. 31292109 10.1136/bmj.l4340

[2] Guarga L, et al. Implementing reflective multicriteria decision analysis (MCDA) to assess orphan drugs value in the Catalan health service (CatSalut). Orphanet J Rare Dis. 2019;14(1):157. 31248421 10.1186/s13023-019-1121-6 PMC6598260

[3] Angelis A, et al. Multiple criteria decision analysis in the context of health technology assessment: a simulation exercise on metastatic colorectal cancer with multiple stakeholders in the English setting. BMC Med Inform Decis Mak. 2017;17(1):149. 29073892 10.1186/s12911-017-0524-3 PMC5658981

[4] de Andrés-Nogales F, et al. A multiple stakeholder multicriteria decision analysis in diabetic macular Edema management: the MULTIDEX-EMD study. Pharmacoecon Open. 2020;4(4):615–624. 32100249 10.1007/s41669-020-00201-2 PMC7688881

[5] Costa CABE, De Corte J, Vansnick J. Macbeth. Int J Inf Tech Decis. 2012;11:359–387.

[6] Costa CABE, De Corte J, Vansnick J. On the mathematical foundations of MACBETH. In: Greco S, Ehrgott M, Figueira RJ, editors. Multiple criteria decision analysis: State of the art surveys. New York (NY): Springer; 2016. p. 421–463.

[7] Bana E, Costa CA, et al. A socio-technical approach for group decision support in public strategic planning: the Pernambuco PPA case. Group Decis Negot. 2014;23(1):5–29.

[8] Greco S, Ehrogott M, Figueira JR. Multiple criteria decision analysis: State of the art surveys. New York: Springer; 2016.

[9] Sanchez-Lopez R, Bana E Costa CA, De Baets B. The MACBETH approach for multi-criteria evaluation of development projects on cross-cutting issues. Ann Oper Res. 2012;199(1):393–408.

[10] Liu W, et al. Burden of lymphoma in China, 1990-2019: an analysis of the global burden of diseases, injuries, and risk factors study 2019. Aging (Albany NY). 2022;14(7):3175–3190. 35398840 10.18632/aging.204006 PMC9037266

[11] Marsh K, et al. Multiple criteria decision analysis for health care decision making--emerging good practices: Report 2 of the ISPOR MCDA emerging good practices task force. Value Health. 2016;19(2):125–137. 27021745 10.1016/j.jval.2015.12.016

[12] Thokala P, et al. Multiple criteria decision analysis for health care decision making--an introduction: Report 1 of the ISPOR MCDA emerging good practices task force. Value Health. 2016;19(1):1–13. 26797229 10.1016/j.jval.2015.12.003

[13] Schnipper LE, et al. American Society of Clinical Oncology statement: a conceptual framework to assess the value of cancer treatment options. J Clin Oncol. 2015;33(23):2563–2577. 26101248 10.1200/JCO.2015.61.6706 PMC5015427

[14] Network, N.C.C. NCCN clinical practice guidelines in oncology (NCCN guidelines) with NCCN evidence blocks™ version 2016. NCCN: America; 2016.

[15] Cherny NI, et al. ESMO – magnitude of clinical benefit scale V.1.0 questions and answers. ESMO Open. 2016;1(5):e000100. 27900206 10.1136/esmoopen-2016-000100 PMC5115817

[16] Wang Susu ZF. Application of the DrugAbacus method in the pricing of anti-cancer drugs. Chin Pharm J. 2019;20(54):1715–1719.

[17] Center, M.S.K.C. Drug Pricing Lab. Methods. 2015.

[18] Health, C.A.F.D. Procedures for the CADTH pan-Canadian oncology drug review. Canada; 2020.

