# Supplementary material for: A value framework for lymphoma therapies based on MACBETH method
Source: Int J Technol Assess Health Care. 2025 May 19;41(1):e49. doi: 10.1017/S0266462325000169 (PMC12322853; doi:10.1017/S0266462325000169)
Supplement: He et al. supplementary material [file S0266462325000169sup001.docx]

**Appendix 1** **An example of the search strategy used in PubMed**

#1 value assessment frameworks [Title/Abstract]

#2 value frameworks [Title/Abstract]

#3 value-based [Title/Abstract]

#4 MCDA [Title/Abstract]

#5 #1 OR #2 OR #3 OR #4

#6 oncology [Title/Abstract]

#7 cancer drugs [Title/Abstract]

#8 anticancer drugs [Title/Abstract]

#9 carcinoma [Title/Abstract]

#10 Chinese caterpillar fungus [Title/Abstract]

#11 lymphoma [Title/Abstract]

#12 #6 OR #7 OR #8 OR #9 OR #10 OR #11

#13 #5 OR #12

**Appendix 2 questionnaire for criteria selection**

1、 Please check "√" before the option that matches your profession

Clinical Experts

Pharmacy Experts

Medical Insurance Experts

Health Economics Experts

2、 Please rate the importance of each criterion, and mark a "√" in the appropriate level of importance , with 0 being the least important and 5 being the most important.

Table 1 Importance Scores for Criteria

| **Value dimension** | **Criteria** | **Importance** | | | | | |
| --- | --- | --- | --- | --- | --- | --- | --- |
|  |  | **0** | **1** | **2** | **3** | **4** | **5** |
| **Clinical**  **value** | Median Overall survival (mOS) |  |  |  |  |  |  |
|  | Median Progression free survival (mPFS) |  |  |  |  |  |  |
|  | Objective response rate |  |  |  |  |  |  |
|  | Complete response |  |  |  |  |  |  |
|  | Duration of response |  |  |  |  |  |  |
|  | Tail of the Curve |  |  |  |  |  |  |
|  | Incidence of adverse events (Grade 1-2) |  |  |  |  |  |  |
|  | Incidence of serious adverse events(Grade3-4) |  |  |  |  |  |  |
|  | Treatment discontinuation rate due to adverse events |  |  |  |  |  |  |
|  | Severity of disease |  |  |  |  |  |  |
|  | Sequence of clinical treatments |  |  |  |  |  |  |
| **Economic**  **value** | Annual direct costs |  |  |  |  |  |  |
|  | Cost-utility |  |  |  |  |  |  |
|  | Budget impact |  |  |  |  |  |  |
| **Patient**  **value** | Health-related quality of life |  |  |  |  |  |  |
|  | Improvement in tumor-related symptoms |  |  |  |  |  |  |
|  | Treatment-free interval |  |  |  |  |  |  |
|  | Changes in drug delivery modalities |  |  |  |  |  |  |
| **Innovation**  **value** | Innovations in therapeutic mechanisms |  |  |  |  |  |  |
|  | Clinical irreplaceability |  |  |  |  |  |  |
|  | Unmet clinical needs |  |  |  |  |  |  |
| **Social**  **value** | Equity |  |  |  |  |  |  |
|  | Increase in social productivity |  |  |  |  |  |  |
|  | Prevalence |  |  |  |  |  |  |
|  | Burden on caregivers |  |  |  |  |  |  |

**Definition of Criteria**

| **Value dimension** | **Criteria** | **Definition or key considerations** |
| --- | --- | --- |

| **Clinical**  **value** | Median Overall survival (mOS) | Period of time between the start of randomization and death due to any cause. | |
| --- | --- | --- | --- |
|  | Median Progression free survival (mPFS) | The period of time between the start of a patient's treatment and the observation of disease progression or the occurrence of death due to any cause. | |
|  | Objective response rate | The proportion of patients whose tumor volume has shrunk to a pre-specified value and who are able to maintain the minimum timeframe required. Objective remission rate generally defined as the sum of complete remission plus partial remission. | |
|  | Complete response | All tumor lesions have disappeared and no tumor tissue can be found in the patient's body, while the patient's tumor marker levels have returned to normal. | |
|  | Duration of response | The period of time between the first assessment of tumor remission or and the first assessment of progressive disease (PD) or death from any cause. | |
|  | Tail of the Curve | When the observation period of a clinical trial ends, a longer plateau in the patient survival curve at a higher survival rate means that the patient has a potentially longer survival. |  |
|  | Incidence of adverse events (Grade 1-2) | Minor Adverse Event |  |
|  | Incidence of serious adverse events(Grade3-4) | Serious Adverse Event |  |
|  | Treatment discontinuation rate due to adverse events | The probability that a patient's treatment will be interrupted due to an adverse event. |  |
|  | Severity of disease | Degree to disease severity or life-threatening, primarily measured by mortality or lethality. The greater the severity of the disease, the greater the health hazard and the greater the clinical value of the drug used to treat it. |  |
|  | Sequence of clinical treatments | The priority of selection and use of treatment options. For example, drugs for first-line treatment are usually the clinically preferred drugs with the most desirable efficacy and relative economy, thus have a higher clinical value. |  |

| **Economic**  **value** | Annual direct costs | Costs directly incurred in healthcare delivery activities, including direct medical costs and direct non-medical costs. |
| --- | --- | --- |
|  | Cost-utility | Intervention options are evaluated economically using monetary and quality-adjusted life-years (QALYs) as indicators of costs and health outputs respectively. ICER (RMB/QALY) usually be used. |
|  | Budget impact | Impact on the health insurance fund when the drug enters the health insurance catalogue. |
| **Patient**  **value** | Health-related quality of life | Refers to an individual's state of an individual's health as influenced by illness, injuries, medical interventions, and aging, as well as his or her personal values and socioeconomic background. The greater the degree to which a disease affects a patient's quality of life, the greater the value of a therapeutic drug or program. |
|  | Improvement in tumor-related symptoms | Improvement of tumor-related symptoms such as pain and malaise compared to existing treatments. |
|  | Treatment-free interval | Extension of treatment-free intervals improves treatment adherence and reduces patient pain with treatment. |
|  | Changes in drug delivery modalities | For example, changing from injection to oral administration reduces patient treatment pain and improves treatment adherence. |
| **Innovation**  **value** | Innovations in therapeutic mechanisms | Drugs with breakthrough improvements in therapeutic mechanisms (e.g., new targets of action), etc., resulting in a major breakthrough in health benefits to patients. |
|  | Clinical irreplaceability | Drugs that are not substitutable in the disease area they treat. In general, drugs with clinical irreplaceability have a higher value. |
|  | Unmet clinical needs | Diseases for which no effective treatment exists are considered to be of high value if the drug is used to treat such diseases in a way that meets a real clinical need. |

| **Social**  **value** | Equity | Impact of new treatments on the health of vulnerable or marginalized populations. |
| --- | --- | --- |
|  | Increase in social productivity | Improved health and reduced healthcare burdens result in patients returning to work, increased efficiency and, in turn, increased productivity in society. |
|  | Prevalence | The frequency with which a particular group of people is currently suffering from a disease during the survey period. All other factors being equal, the more prevalent a disease is, the greater the harm it causes society, and the greater the value of medication for its treatment. |
|  | Burden on caregivers | Refers to the negative emotional changes felt by the carer and the physical, mental, emotional, social and financial costs as a result of caring for the patient. |

**Appendix 3 Questionnaire for the value function**

The evaluation criteria included in the value framework of this study include: (1) median progression-free survival (mPFS); (2) objective remission rate (ORR); (3) incidence of serious adverse events(Grade 3-4) (≥3 AE); (4) treatment discontinuation rate due to AE (AE-TDR); (5) quality-adjusted life years (QALYs); (6) annual direct medical costs (ADMC); (7) dosage and administration; (8) mortality of disease; (9) The number of alternative medicines with the same indication and mechanism

- Instructions for completion:

1. The first part of the questionnaire consists of 9 questions corresponding to the 9 criteria, please compare the value differences between the different attribute levels of each criterion two by two;
2. The magnitude of the difference in value between the different levels is differentiated according to seven levels set by the MACBETH method: no difference (no), very weak difference (very weak), weak difference (weak), moderate difference (moderate), strong difference (strong), very strong difference (very strong), and extreme difference (extreme);
3. The difference in value of a comparison between levels can be the same;
4. The difference in value of the comparison between two levels can be between two neighboring levels of difference. e.g. the difference of the comparison between two levels can be: difference very weak ~ difference weaker;
5. For details, please refer to the example below.

Take Overall Survival (OS) as an example: Assuming that there are 5 reference levels of OS, 24 months, 18 months, 12 months, 8 months, and 4 months, the difference in value between the levels is compared as shown in the following box plot.

| **OS** | **24 months** | **18 months** | **12 months** | **8 months** | **4 months** |
| --- | --- | --- | --- | --- | --- |
| **24 months** | **No** | **1 ^a^** | **2 ^b^** | **4** | **5** |
| **18 months** |  | **No** | **2 ^c^** | **3-4 ^d^** | **4-5** |
| **12 months** |  |  | **No** | **2** | **3** |
| **8 months** |  |  |  | **No** | **2-3** |
| **4 months** |  |  |  |  | **No** |

| **6** | **Extreme** |
| --- | --- |
| **5** | **Very Strong** |
| **4** | **Strong** |
| **3** | **Moderate** |
| **2** | **Weak** |
| **1** | **Very weak** |
| **0** | **No** |

- **Requirements：**Use the contents of the cells in the first column to compare the contents of the cells in the first row in turn. Fill in the white cells above "no difference" with the number representing the level of the difference.
- **Legend：**

a indicates that the filler believes that the difference between the value with an OS of 24 months and the value with an OS of 18 months is very weak;

b indicates that the difference between the value with an OS of 24 months and the value with an OS of 12 months was perceived by the filler to be weak;

c indicates that the filler believes that the difference is also weak for a value with an OS of 18 months compared to a value with an OS of 12 months;

d indicates that the filler believes that the difference between value with an OS of 18 months compared to value with an OS of 8 months is between a moderate difference ~ a strong difference.

**3 Comparison of the difference in value between different levels**

**3.1 Median progression-free survival（mPFS）**

**Question 1: In your opinion, for lymphoma therapeutics, what is the difference between two-by-two comparisons of each value?**

Please refer to the example instructions and enter the number representing the

magnitude of the difference in the table below, only the white cell is required.

| **mPFS** | **4** | **5** | **17** | **41*** | **49** |
| --- | --- | --- | --- | --- | --- |
| **4** | **No** |  |  |  |  |
| **5** |  | **No** |  |  |  |
| **17** |  |  | **No** |  |  |
| **41** |  |  |  | **No** |  |
| **49** |  |  |  |  | **No** |

| **6** | **Extreme** |
| --- | --- |
| **5** | **Very Strong** |
| **4** | **Strong** |
| **3** | **Moderate** |
| **2** | **Weak** |
| **1** | **Very weak** |
| **0** | **No** |

*Note: In some clinical trials, mPFS is not achieved, and for survival curves where mPFS is not achieved, mPFS is calculated by extrapolation using a best-fit survival model. For example, an mPFS of 41 months is derived from extrapolating a PFS curve from a clinical trial of obrutinib in relapsed or refractory chronic lymphocytic leukaemia/small lymphocytic lymphoma.

**3.2 Quality-adjusted life years（QALYs）**

**Question 2: In your opinion, for lymphoma treatment drugs, when their QALYs (in years) to patients are the values presented in the table below, what is the difference between the two-by-two comparisons of each value?**

Please refer to the example instructions and fill in the numbers representing the magnitude of the difference in the table below, only the white cells are required.

| **QALYs** | **1.4** | **1.8** | **5.2** | **9.6** | **11.5** |
| --- | --- | --- | --- | --- | --- |
| **1.4** | **No** |  |  |  |  |
| **1.8** |  | **No** |  |  |  |
| **5.2** |  |  | **No** |  |  |
| **9.6** |  |  |  | **No** |  |
| **11.5** |  |  |  |  | **No** |

| **6** | **Extreme** |
| --- | --- |
| **5** | **Very Strong** |
| **4** | **Strong** |
| **3** | **Moderate** |
| **2** | **Weak** |
| **1** | **Very weak** |
| **0** | **No** |

**3.3 Objective response rate（ORR）**

**Question 3: In your opinion, what is the difference between the two comparisons for a lymphoma drug when the ORR for patients is the value presented in the table below?**

Please refer to the example instructions and enter the number representing the magnitude of the difference in the table below, only the white cell is required.

| **ORR** | **28%** | **35%** | **79%** | **93%** | **100%** |
| --- | --- | --- | --- | --- | --- |
| **28%** | **No** |  |  |  |  |
| **35%** |  | **No** |  |  |  |
| **79%** |  |  | **No** |  |  |
| **93%** |  |  |  | **No** |  |
| **100%** |  |  |  |  | **No** |

| **6** | **Extreme** |
| --- | --- |
| **5** | **Very Strong** |
| **4** | **Strong** |
| **3** | **Moderate** |
| **2** | **Weak** |
| **1** | **Very weak** |
| **0** | **No** |

**3.4 Incidence of serious adverse events(Grade3-4)（≥3 AE）**

**Question 4: In your opinion, what is the difference between the two comparisons for lymphoma therapeutic agents when the incidence of grade 3 or higher adverse events for patients is the value presented in the table below?**

Please refer to the example instructions and enter the number representing the magnitude of the difference in the table below, only the white cell is required.

| **≥3 AE** | **100%** | **91%** | **58%** | **21%** | **17%** |
| --- | --- | --- | --- | --- | --- |
| **100%** | **No** |  |  |  |  |
| **91%** |  | **No** |  |  |  |
| **58%** |  |  | **No** |  |  |
| **21%** |  |  |  | **No** |  |
| **17%** |  |  |  |  | **No** |

| **6** | **Extreme** |
| --- | --- |
| **5** | **Very Strong** |
| **4** | **Strong** |
| **3** | **Moderate** |
| **2** | **Weak** |
| **1** | **Very weak** |
| **0** | **No** |

**3.5 Treatment discontinuation rate due to adverse events（AE-TDR）**

**Question 5: In your opinion, for lymphoma drugs, what is the difference between the two comparisons of the values presented in the table below when the rate of discontinuation of treatment due to adverse events during treatment is the value presented in the table below?**

Please refer to the example and fill in the numbers representing the magnitude of the difference in the table below, only the white cell is required.

| **AE-TDR** | **30%** | **25%** | **10%** | **3%** | **2%** |
| --- | --- | --- | --- | --- | --- |
| **30%** | **No** |  |  |  |  |
| **25%** |  | **No** |  |  |  |
| **10%** |  |  | **No** |  |  |
| **3%** |  |  |  | **No** |  |
| **2%** |  |  |  |  | **No** |

| **6** | **Extreme** |
| --- | --- |
| **5** | **Very Strong** |
| **4** | **Strong** |
| **3** | **Moderate** |
| **2** | **Weak** |
| **1** | **Very weak** |
| **0** | **No** |

**3.6 Annual direct medical costs**

**Question 6: In your opinion, for a lymphoma drug, what is the difference in value between the two comparisons for the patient's annual direct medical costs (in millions of dollars) for the lymphoma drug when the values are presented in the table below?**

Please refer to the example instructions and enter the number representing the magnitude of the difference in the table below, only the white cell is required.

| **Annual direct costs** | **12.8** | **16.0** | **20.0** | **39.0** | **46.8** |
| --- | --- | --- | --- | --- | --- |
| **12.8** | **No** |  |  |  |  |
| **16.0** |  | **No** |  |  |  |
| **20.0** |  |  | **No** |  |  |
| **39.0** |  |  |  | **No** |  |
| **46.8** |  |  |  |  | **No** |

| **6** | **Extreme** |
| --- | --- |
| **5** | **Very Strong** |
| **4** | **Strong** |
| **3** | **Moderate** |
| **2** | **Weak** |
| **1** | **Very weak** |
| **0** | **No** |

**3.7 dosage and administration**

**Question 7: In your opinion, for lymphoma therapeutics, what is the difference in value of the two-way comparison between the modes of administration when the dosage is as presented in the table below?**

Please refer to the example instructions and enter the number representing the magnitude of the difference in the table below, only the white cell is required.

| **Appropriateness**  **-dosage** | **Oral once daily** | **Oral twice daily** | **Intravenous once every three weeks** | **Intravenous once a fortnight** | **Intravenous, once a week** |  |
| --- | --- | --- | --- | --- | --- | --- |
| **Oral once daily** | **No** |  |  |  |  | |
| **Oral twice daily** |  | **No** |  |  |  | |
| **Intravenous once every three weeks** |  |  | **No** |  |  | |
| **Intravenous once a fortnight** |  |  |  | **No** |  | |
| **Intravenous, once a week** |  |  |  |  | **No** | |

| **6** | **Extreme** |
| --- | --- |
| **5** | **Very Strong** |
| **4** | **Strong** |
| **3** | **Moderate** |
| **2** | **Weak** |
| **1** | **Very weak** |
| **0** | **No** |

**3.8 Mortality of disease**

**Question 8: In your opinion, what is the difference in value between the two comparisons for lymphoma drugs when the disease mortality rate (in 100,000) for the indication is the value presented in the table below?**

Please refer to the example and fill in the numbers representing the magnitude of the difference in the table below, only the white cells are required.

| **Mortality** | **0.15** | **0.19** | **1.32** | **2.45** | **2.94** |
| --- | --- | --- | --- | --- | --- |
| **0.15** | **No** |  |  |  |  |
| **0.19** |  | **No** |  |  |  |
| **1.32** |  |  | **No** |  |  |
| **2.45** |  |  |  | **No** |  |
| **2.94** |  |  |  |  | **No** |

| **6** | **Extreme** |
| --- | --- |
| **5** | **Very Strong** |
| **4** | **Strong** |
| **3** | **Moderate** |
| **2** | **Weak** |
| **1** | **Very weak** |
| **0** | **No** |

**3.9 The number of alternative medicines with the same indication and mechanism**

**Question 9: In your opinion, for a lymphoma drug, when the number of substitutes (in units) for the drug (with the same indication and the same mechanism of action) is the value presented in the table below, what is the difference in value for a two-by-two comparison of each value?**

Please fill in the table below with the numbers representing the magnitude of the difference, referring to the example instructions, and only fill in the white cells.

| **Innovativeness** | **0** | **1** | **2** | **3** | **4** |
| --- | --- | --- | --- | --- | --- |
| **0** | **No** |  |  |  |  |
| **1** |  | **No** |  |  |  |
| **2** |  |  | **No** |  |  |
| **3** |  |  |  | **No** |  |
| **4** |  |  |  |  | **No** |

| **6** | **Extreme** |
| --- | --- |
| **5** | **Very Strong** |
| **4** | **Strong** |
| **3** | **Moderate** |
| **2** | **Weak** |
| **1** | **Very weak** |
| **0** | **No** |

**Appendix 4 Hypothetical alternatives**

| Alternatives  Attributes | [all lower] | [mPFS] | [QALYs] | $\left[ ORR \right]$ | [≥3AE] | [AE_TDR] | [ADMC] | [Admin] | [Mortality] | [Quantity] |
| --- | --- | --- | --- | --- | --- | --- | --- | --- | --- | --- |

| mPFS（month） | 5 | 41 | 5 | 5 | 5 | 5 | 5 | 5 | 5 | 5 |
| --- | --- | --- | --- | --- | --- | --- | --- | --- | --- | --- |

| QALYs | 1.8 | 1.8 | 9.6 | 1.8 | 1.8 | 1.8 | 1.8 | 1.8 | 1.8 | 1.8 |  |
| --- | --- | --- | --- | --- | --- | --- | --- | --- | --- | --- | --- |
| ORR | 35% | 35% | 35% | 93% | 35% | 35% | 35% | 35% | 35% | 35% | |
| ≥3 AE | 91% | 91% | 91% | 91% | 21% | 91% | 91% | 91% | 91% | 91% | |
| AE-TDR | 25% | 25% | 25% | 25% | 25% | 3% | 25% | 25% | 25% | 25% | |
| ADMC(RMB) | 39 | 39 | 39 | 39 | 39 | 39 | 16 | 39 | 39 | 39 | |
| Dosage and administration | iv.q2w | iv.q2w | iv.q2w | iv.q2w | iv.q2w | iv.q2w | iv.q2w | po.bid | iv.q2w | iv.q2w | |
| Mortality  （per 100,000） | 0.19 | 0.19 | 0.19 | 0.19 | 0.19 | 0.19 | 0.19 | 0.19 | 2.45 | 0.19 | |
| The number of alternative medicines | 3 | 3 | 3 | 3 | 3 | 3 | 3 | 3 | 3 | 1 | |

iv.q2w: Intravenous injection, every two weeks; po.bid: Oral, twice a day

**Appendix 5 Importance Scores and Selection Reasons of Criteria**

Guidelines with an expert importance score of ≤3.5 are first excluded as less important, and the remaining guidelines are decided to be included or not based on the order of the six guideline selection principles - relevant literature - expert opinion proposed by the ISPOR guidelines, respectively. Inclusion reasons for criteria are shown detailed in the following table.

| **Criteria** | **Mean Scores** | **Whether to be included** | **Reasons for exclusion and other specification** |
| --- | --- | --- | --- |
| Median overall survival, mOS | 4.7 | No | (1) A systematic review of pivotal clinical trials of lymphoma therapeutics marketed in China from 2017 to present reveals that none of the clinical trials used OS as the primary efficacy indicator, and the vast majority of them fail to achieve mOS; (2) ISPOR suggests that comprehensive indicators should be selected as much as possible and QALY is a comprehensive indicator which reveals survival time and quality of life , QALY is included instead of OS and health-related quality of life. |
| annual direct costs | 4.7 | Yes | Due to poor availability of direct non-medical cost data, we use annual direct medical costs as a substitution. |
| Health-related quality of life | 4.7 | Yes | Take data availability into account, QALY is used instead. |
| Improvement in tumor-related symptoms | 4.6 | No | Data on this metric are rare and not available. |
| Clinical irreplaceability | 4.5 | Yes | Measured by the number of alternative medicines with the same indication and mechanism. |
| Median progression free survival | 4.5 | Yes | - |
| Treatment discontinuation rate due to adverse events | 4.5 | Yes | - |
| Objective response rate (ORR) | 4.4 | Yes | - |
| Complete response (CR) | 4.3 | No | Few trials use CR as a primary efficacy indicator, besides CR is part of ORR, violating the principle of non-redundancy. |
| Cost-Utility | 4.3 | No | It is not easy to compare multiple options based on ICER values, and ISPOR states that CUA already includes cost and QALY, so it is against the principle of redundancy to include them all. |
| Duration of response (DOR) | 4.2 | No | Few clinical trials use this indicator as the primary efficacy indicator |
| Incidence of grade serious adverse events(Grade3-4) | 4.2 | Yes | - |
| Unmet clinical needs | 4.2 | No | Lack of uniform definition and quantitative evaluation methods for unmet clinical needs, which violates the principle of actionability/clustering. |
| Disease severity | 4.1 | Yes | Measured by disease mortality, for which data are available. |
| Treatment-free interval | 4.0 | No | Few clinical trials report this data, which is contrary to the principle of actionability. |
| Tail of the Curve | 3.9 | No | High uncertainty, in addition to the trailing effect of drug efficacy, it varies according to the length of trial follow-up, sample size, and treatment after progression. |
| Innovations in therapeutic mechanisms | 3.7 | No | Expert think that innovations in therapeutic mechanisms are of little significance if they do not lead to innovations in health benefits, which are already reflected in efficacy and safety indicators. Besides, innovations in therapeutic mechanisms is difficult to quantified. |
| Changes in drug delivery modalities | 3.6 | Yes | Experts believe frequency of administration should also be taken into account. ISPOR suggests that the mode of administration and frequency of administration should be combined into one criterion. Therefore, we adjusted this criterion as Dosage and administration**.** |
| Sequence of clinical treatments | 3.5 | No | Mean expert importance score was not more than 3.5 points. |
| Budget Impact | 3.5 | No | Mean expert importance score was not more than 3.5 points. In addition, direct healthcare cost which may also reflect the impact on the healthcare has been included. |
| Prevalence | 3.5 | No | Mean expert importance score was not more than 3.5 points. |
| Burden on caregivers | 3.5 | No | Mean expert importance score was not more than 3.5 points. |
| Equity | 3.4 | No | Mean expert importance score was not more than 3.5 points. |
| Increase in social productivity | 3.3 | No | Mean expert importance score was not more than 3.5 points. |
| Incidence of adverse events(Grade1-2) | 3.0 | No | Mean expert importance score was not more than 3.5 points. |

**Appendix 6 Piecewise linear value function of QALYs, ORR, serious adverse events (grade 3-4), AE-TDR, ADMC, Feasibility, Severity of disease and Innovation**


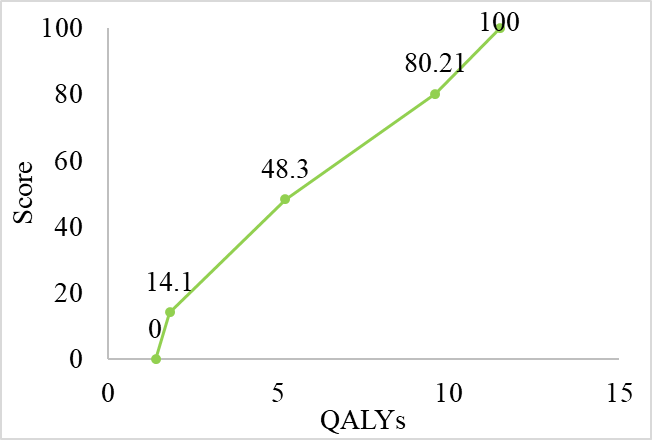
F**igure 6.1 Value function of QALYs**

$$V_{QALYs}\left( x \right)=\left\{ \begin{aligned} 10.42x-19.78, 9.6<x\leq11.5 \\ 7.25x+10.59, 5.2<x\leq9.6 \\ 10.06x-4.01, 1.8<x\leq5.2 \\ 35.25x-49.35, 1.4<x\leq1.8 \end{aligned} \right.$$

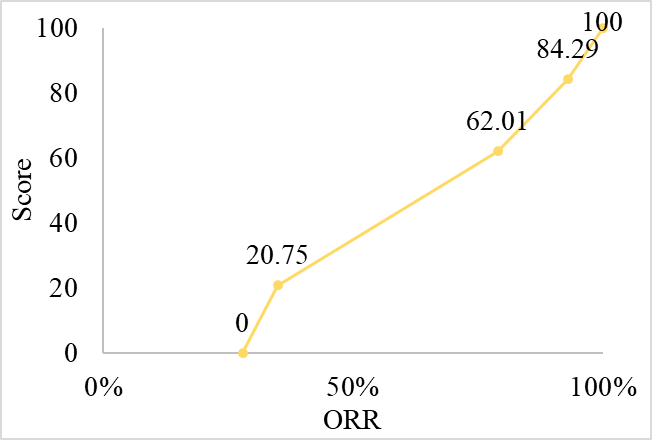
**Figure 6.2 Value function of ORR**

$$V_{ORR}\left( x \right)=\left\{ \begin{aligned} 224.43x-124.43, 93\%<x\leq100\% \\ 159.14x-63.71, 79\%<x\leq93\% \\ 93.77x-12.07, 35\%<x\leq79\% \\ 296.43x-83.00, 28\%<x\leq35\% \end{aligned} \right.$$

**Figure 6.3 Value function of serious adverse events**

**
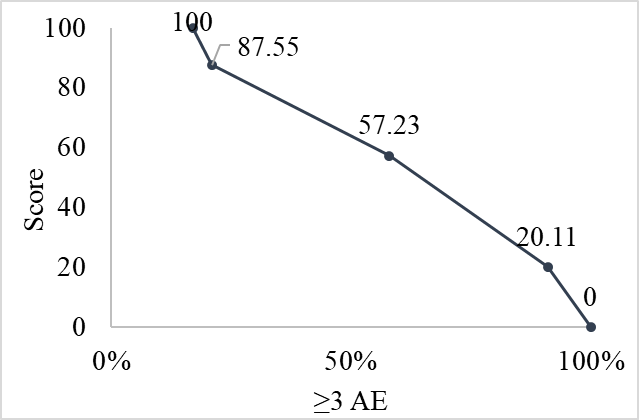
**

$$V_{\geq3AE}\left( x \right)=\left\{ \begin{aligned} -311.25x+152.91, 0.17<x\leq0.21 \\ -81.95x+104.76, 0.21<x\leq0.58 \\ -112.48x+122.47, 0.58<x\leq0.91 \\ -223.44x+223.44, 0.91<x\leq1 \end{aligned} \right.$$

**
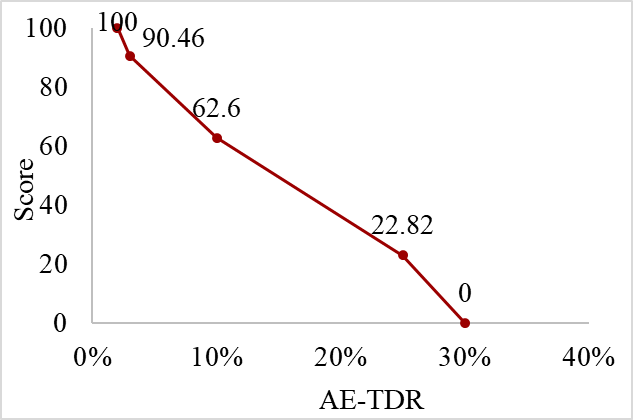
Figure 6.4 Value function of AE-TDR**

$$V_{AE_{TDR}}\left( x \right)=\left\{ \begin{aligned} -954.00x+119.08, 0.02<x\leq0.03 \\ -398.00x+102.40, 0.03<x\leq0.1 \\ -265.20x+89.12, 0.1<x\leq0.25 \\ -456.40x+136.92, 0.25<x\leq0.3 \end{aligned} \right.$$

**
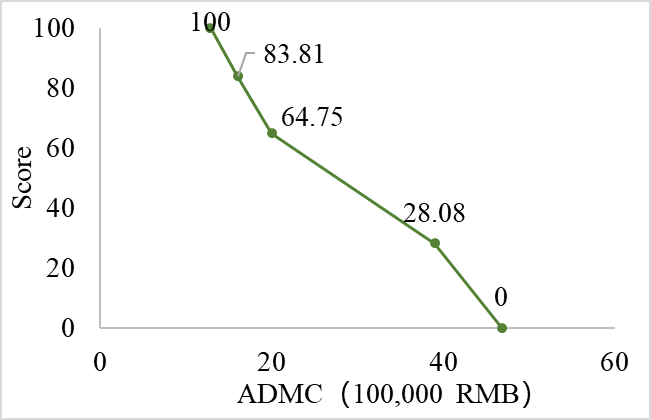
Figure 6.5 Value function of ADMC**

$$V_{ADMC}\left( x \right)=\left\{ \begin{aligned} -5.06x+164.76, 12.8<x\leq16 \\ -4.77x+160.05, 16<x\leq20 \\ -1.93x+103.35, 20<x\leq39 \\ -3.60x+168.48, 39<x\leq46.8 \end{aligned} \right.$$

**
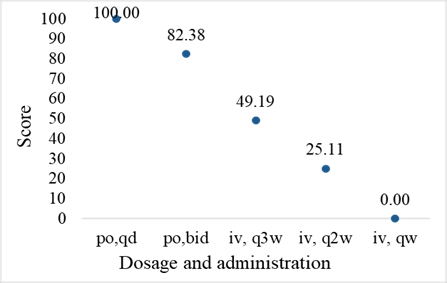
Figure 6.6 Value function of dosage and administration**

$$V_{Admin}\left( x \right)=\left\{ \begin{aligned} 100, x=po,qd \\ 82.38, x=po,bid \\ 49.19, x=iv, q3w \\ 25.11, x=iv, q2w \\ 0, x=iv, qw \end{aligned} \right.$$

**
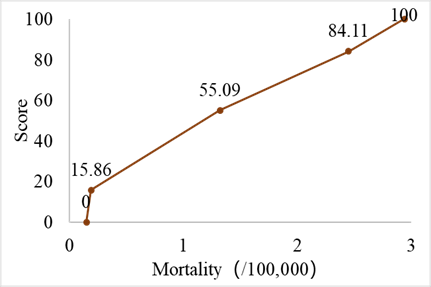
Figure 6.7 Value function of mortality**

$$V_{Mortality}\left( x \right)=\left\{ \begin{aligned} 32.43x+4.66, 2.94<x\leq2.45 \\ 25.68x+21.19, 2.45<x\leq1.32 \\ 34.72x+9.26, 1.32<x\leq0.19 \\ 396.50x-59.48, 0.19<x\leq0.15 \end{aligned} \right.$$

**
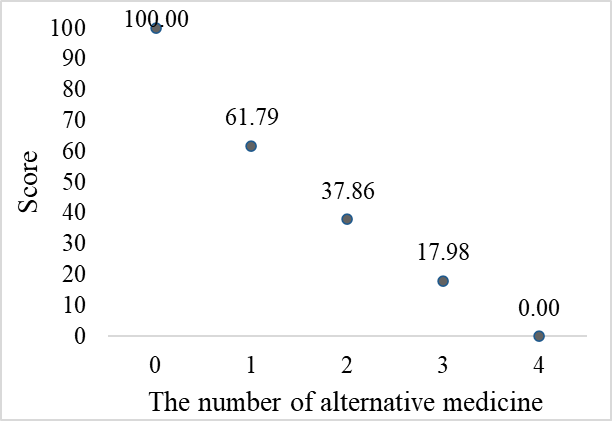
Figure 6.8 Value function of the number of alternative medicine**

$$V_{Quantity}\left( x \right)=\left\{ \begin{aligned} 100, x=0 \\ 61.79, x=1 \\ 37.86, x=2 \\ 17.98, x=3 \\ 0, x=4 \end{aligned} \right.$$
